# The Law Relating to Infectious Diseases.—II

**Published:** 1902-08-02

**Authors:** J. E. R. Stephens

**Affiliations:** Barrister-at-law, Author of "Digest of Public Health Cases," etc. etc.


### The Institutional Workshop.

### THE LAW RELATING TO INFECTIOUS DISEASES.—II.

By J. E. R. STEPHENS, Barrister-at-law, Author of "Digest of Public Health Cases," etc. etc.

### AMBULANCES.

By sect. 123 of the Public Health Act, 1875, any local authority may provide and maintain a carriage or carriages suitable for the conveyance of persons suffering under any infectious disorder, and may pay the expense of conveying therein any person so suffering to a hospital or other place of destination. A memorandum on "ambulances" was issued by the Local Government Board in December, 1876. It is as follows:—

For the conveyance of patients who are sick with infectious disease, special carriages, which are known by the name of "ambulances," are necessary. Such carriages may be provided by sanitary authorities under sect. 123 of the Public Health Act, 1875. The following points have to be attended to in the provision and use of such carriages:—(1) If the ambulance be intended only for journeys of not more than a mile, it may be made so as to be carried between two people, or it may be on wheels and to be drawn by hand. If a distance be above a mile the ambulance should be drawn by a horse. Every ambulance on wheels should have easy carriage-springs. (2) In the construction of an ambulance, special regard should be had to the fact that after each use it has to be cleansed and disinfected. The entire interior, and the bed-frame and bed, should be of materials that can be washed. (3) The ambulance should be such that the patient can lie full length in it, and the bed-frame and bed should be movable, so that the patient can be arranged upon the bed before being taken out of his house. (4) With an ambulance there should always be a person specially in charge of the patient, and a horse ambulance should have a seat for such person inside the carriage. (5) After every use of an ambulance for infectious disease, it should be cleansed and disinfected to the satisfaction of a medical officer. (6) Both in very populous districts, and in districts which are of very wide area, it may often happen that more than one ambulance will be wanted at one time; and, in any district, if more than one infectious disease is prevailing, there will be an evident sanitary advantage in having more than one ambulance for use.

### BEDDING.

By sect. 121 of the Act of 1875 any local authority may direct the destruction of any bedding, clothing, or other articles which have been exposed to infection from any dangerous infectious disorder, and may give compensation for the same. And by sect. 122 any local authority may provide a proper place, with all necessary apparatus and attendance, for the disinfection of bedding, clothing, or other articles which have become infected, and may cause any articles brought for disinfection to be disinfected free of charge.

Where the Infectious Diseases (Prevention) Act, 1890 has been adopted any local authority, or the medical officer of health of any local authority generally empowered by the authority in that behalf, may by notice in writing require the owner of any bedding, clothing, or other articles which have been exposed to the infection of any infectious disease to cause the same to be delivered over to an officer of the local authority for removal for the purpose of disinfection; and any person who fails to comply with such a requirement shall be liable to a penalty not exceeding £10. The bedding, clothing, and articles shall be disinfected by the authority, and shall be brought back and delivered to the owner free of charge, and if any of them suffer any unnecessary damage the authority shall compensate the owner for the same and the amount of compensation shall be recoverable in, and in case of dispute shall be settled by, a court of summary jurisdiction (sect. 6).

Sect. 308 of the Public Health Act, 1875, provides that where any person sustains damage, by reason of the exercise of any of the powers of this Act, in relation to any matter as to which he is not himself in default, full compensation shall be made to such person by the local authority exercising such powers, and any dispute as to the fact of damage or amount of compensation shall be settled by arbitration in manner provided by this Act, or if the compensation claimed does not exceed the sum of £20, the same may at the option of either party be ascertained by, and recovered before, a court of summary jurisdiction. Under sect. 121, the local authority appear to have a discretion, and not to be obliged to give compensation if they consider that the circumstances do not entitle the claimant to any.

### REMOVAL OF DEAD BODY.

Sect. 142 of the Public Health Act, 1875, enacts that where the body of one who has died of any infectious disease is retained in a room in which persons live or sleep, or any dead body which is in such a state as to endanger the health of the inmates of the same house or room is retained in such house or room, any justice may, on a certificate signed by a legally qualified medical practitioner, order the body to be removed, at the cost of the local authority, to any mortuary provided by such authority, and direct the same to be buried within a time to be limited in such order; and unless the friends or relations of the deceased undertake to bury the body within the time so limited, and do bury the same, it shall be the duty of the relieving officer to bury such body at the expense of the poor-rate, but any expense so incurred may be recovered by the relieving officer in a summary manner from any person legally liable to pay the expense of such burial. Any person obstructing the execution of an order made by a justice under this section shall be liable to a penalty not exceeding £5.

Where the local authority have provided a mortuary, this section applies; but where the Infectious Diseases (Prevention) Act, 1890, has been adopted, the justice may order the body to be removed to any available mortuary. The sections of the Act of 1890 relating to the bodies of persons who have died from infectious disease are 8 to 11.

Section 8 provides that no person without the sanction in writing of the medical officer of health or of a registered medical practitioner, shall retain unburied elsewhere than in a public mortuary or in a room not used at the time as a dwelling-place, sleeping-place, or workroom, for more than 48 hours, the body of any person who has died of any infectious disease.

The principles of the common law with respect to the right of burial were laid down by Lord Denman, C.J., as follows:—"Every person dying in this country, and not within certain exclusions laid down by the ecclesiastical law, has a right to Christian burial; and that implies the right to be carried from the place where his body lies to the parish cemetery. Further, to use the words of Lord Stowell (*Gilbert* v. *Buzzard*, 2 Hagg. Consist. Rep. 333), 'that bodies should be carried in a state of naked exposure to the grave would be a real offence to the living, as well as an apparent indignity to the dead.' We have no doubt, therefore, that the common law casts on someone the duty of carrying to the grave, decently covered, the dead body of any person dying in such a state of indigence as to leave no funds for that purpose. The feelings and the interests of the living require this, and create the duty. . . . It should seem that the individual under whose roof a poor person dies is bound to carry the body, decently covered, to the place of burial; he cannot keep him unburied, nor do anything which prevents Christian burial; he cannot, therefore, cast him out, so as to expose the body to violation, or to offend the feelings or endanger the health of the living; and, for the same reason, he cannot carry him uncovered to the grave" (*R.* v. *Stewart*, 12 A. & E. 773). Poor Law guardians are authorised to bury the bodies of any poor persons within their parish or union at the cost of the poor rate; but they are not bound to do so where the body is not lying in the workhouse or on premises belonging to the parish or union.

By sect. 9 of the Act of 1890, it is enacted that if any person shall die from any infectious disease in any hospital or place of temporary accommodation for the sick, and the Medical Officer of Health, or any other registered medical practitioner, certifies that in his opinion it is desirable, in order to prevent the risk of communicating any infectious disease or of spreading infection, that the body shall not be removed from such hospital or place except for the purpose of being forthwith buried, it shall not be lawful for any person or persons to remove such body from such hospital or place except for the last-mentioned purpose; and when the body is taken out of the hospital for such purpose, it shall be forthwith carried or taken direct to some cemetery or place of burial, and shall be forthwith there buried; and any person wilfully offending against this section shall be liable to a penalty not exceeding £10. Nothing in this Act shall prevent the removal of any dead body from any hospital or temporary place of accommodation for the sick to any mortuary, and such mortuary shall, for the purposes of this section, be deemed part of such hospital or place as aforesaid.

By sect. 10, where the body of any person who has died from any infectious disease remains unburied elsewhere than in a mortuary or in a room not used at the time as a dwelling-place, sleeping-place, or work-room, for more than 48 hours after death, without the sanction of the Medical Officer of Health or of a registered medical practitioner, or where the dead body of any person is retained in any house or building so as to endanger the health of the inmates of such house or building, or of any adjoining or neighbouring house or building, any justice may, on the application of the Medical Officer of Health, order the body to be removed at the cost of the local authority to any available mortuary, and direct the same to be buried in the time to be limited in the order; and any justice may, in the case of the body of any person who has died of any infectious disease, or in any case in which he shall consider immediate burial necessary, direct the body to be so buried. Unless the friends or relatives of the deceased undertake to bury, and do bury the body within the time limited by such order, it shall be the duty of the relieving officer of the relief district from which the body has been removed to the mortuary, or in which the body shall be, if it has not been so removed, to bury such body, and any expense so incurred may be charged by the relieving officer in his accounts, and may be recovered by the board of guardians in a summary manner from any person legally liable to pay the expenses of such burial.

Under sect. 142 of the Public Health Act, 1875, a justice of the peace may, in certain cases, order the removal of a dead body to a mortuary. That section enacts that, where the body of one who has died of any infectious disease is retained in a room in which persons live or sleep, or any dead body which is in such a state as to endanger the health of the inmates of the same house or room, is retained in such house or room, any justice may, on a certificate signed by a legally qualified medical practitioner, order the body to be removed, at the cost of the local authority, to any mortuary provided by such authority, and direct the same to be buried within a time to be limited in such order, and unless the friends or relations of the deceased undertake to bury the body within the time so limited, and do bury the same, it shall be the duty of the relieving officer to bury such body at the expense of the poor rate, but any expense so incurred may be recovered by the relieving officer in a summary manner from any person legally liable to pay the expense of such burial. Any person obstructing the execution of an order made by a justice under this section shall be liable to a penalty not exceeding £5.

And by sect. 11 of the Act of 1890 it is enacted that any person who hires or uses a public conveyance, other than a hearse, for the conveyance of the body of a person who has died from any infectious disease, without previously notifying to the owner or driver of such public conveyance that the person whose body is or is intended to be so conveyed has died from infectious disease, and, after any such notification as aforesaid, any owner or driver of a public conveyance, other than a hearse, which has been used for conveying the body of a person who has died from infectious disease, who shall not immediately afterwards provide for the disinfection of such conveyance, shall be guilty of an offence under this Act.

*(To be continued.)*

